# A promising frontier: targeting NETs for stroke treatment breakthroughs

**DOI:** 10.1186/s12964-024-01563-4

**Published:** 2024-04-23

**Authors:** Huijie Fang, Yunfei Bo, Zhongfei Hao, Ge Mang, Jiaqi Jin, Hongjun Wang

**Affiliations:** 1 Department of Neurosurgery, The Second Affiliated Hospital of Harbin Medical University, Harbin, 150001 China; 2 Department of Cardiology, The Second Affiliated Hospital of Harbin Medical University, Harbin, China; 3 Department of Neurosurgery, Xuanwu Hospital, Capital Medical University, Beijing, 100053 China

**Keywords:** Neutrophil extracellular trap, Neutrophil, Ischaemic stroke, t-PA

## Abstract

Stroke is a prevalent global acute cerebrovascular condition, with ischaemic stroke being the most frequently occurring type. After a stroke, neutrophils accumulate in the brain and subsequently generate and release neutrophil extracellular traps (NETs). The accumulation of NETs exacerbates the impairment of the blood‒brain barrier (BBB), hampers neovascularization, induces notable neurological deficits, worsens the prognosis of stroke patients, and can facilitate the occurrence of t-PA-induced cerebral haemorrhage subsequent to ischaemic stroke. Alternative approaches to pharmacological thrombolysis or endovascular thrombectomy are being explored, and targeting NETs is a promising treatment that warrants further investigation.

## Introduction

Stroke is a highly prevalent and debilitating neurological disorder that is associated with significant rates of morbidity, disability, mortality, and recurrence. Cerebral stroke is an acute cerebrovascular disease, the pathogenesis of which is characterized by inadequate blood flow to the brain due to the rupture or occlusion of cerebrovascular vessels, thereby causing brain damage [1]. There are two primary classifications of stroke: ischaemic stroke and haemorrhagic stroke, with the former constituting more than 80% of all cases. Statistical evidence from China indicates that stroke resulted in the deaths of approximately 1.57 million individuals in 2018 [2]. In 2017, there were 1.12 million incident strokes in the European Union, 9.53 million stroke survivors, 0.46 million deaths, and 7.06 million disability-adjusted life years lost because of stroke [3]. More than 795,000 strokes occur in the United States each year. Approximately 610,000 of these strokes were first-time strokes, while 185,000 occurred in patients with previous strokes [4]. Consequently, it is crucial to investigate the fundamental causes of this disease and identify new pharmacological targets to facilitate the advancement of innovative therapeutic interventions and improve patient prognosis. Initially, the recruitment of neutrophils to the brain among various immune cells results in the release of reactive oxygen species (ROS) that exacerbate blood‒brain barrier (BBB) impairment and worsen stroke. Investigations have revealed that neutrophils release NETs when stimulated by substances such as lipopolysaccharide (LPS) [5]. As the study of NETs progressed, researchers observed significantly elevated levels of plasma NETs in individuals with ischaemic stroke, and these higher levels were correlated with inferior neurological function [6].

Composed of DNA structures embellished with diverse cytosolic, granule, and nuclear proteins, NETs effectively capture bacteria, viruses, and fungi. Imbalanced immune responses can have a significant impact on the dysregulated release of NETs, which have dual roles as antimicrobial agents and contributors to heightened inflammation and tissue damage. Consequently, NETs have a vital impact on the progression of different medical conditions, such as stroke. Furthermore, the presence of target neutrophils and NETs has a positive effect on the restructuring of blood vessels in the brain and the restoration of functionality during the later phases after stroke. This paper provides an overview of the structure and occurrence of NETs in relation to cerebral thrombosis and brain injury, aiming to provide insights for improved identification and management of stroke in the future.

## Physiological functions of NETs

### Structure and basic functions of NETs

Historically, neutrophils have been regarded as the primary component of the initial immune response of the innate immune system to microbial invasion [7, 8]. Furthermore, in addition to their ability to eradicate bacteria, the stimulation of neutrophils triggers the release of nuclear and granular contents, culminating in the formation of intricate web-like DNA structures referred to as NETs [5, 9].

Prior to stimulation, naive neutrophils display a spherical morphology with distinct membrane folds. However, following stimulation with interleukin-8 (IL-8), phorbol myristate acetate (PMA), or LPS, neutrophils undergo morphological changes characterized by a flattened shape and the emergence of membrane protrusions. Notably, experimental observations have demonstrated that only activated neutrophils, as opposed to nonstimulated neutrophils, exhibit conspicuous extracellular formations. These formations, known as NETs, are notably fragile and devoid of enclosing membranes.

NETs are composed of chromatin filaments that are covered with histones, enzymes, and different proteins present in granular and cytosolic compartments. Proteins such as neutrophil elastase (NE), cathepsin G, and myeloperoxidase (MPO) are found in azurophilic granules [10, 11], along with proteins from specific and tertiary granules such as lactoferrin and gelatinase. However, proteins such as CD63, which is a granule membrane protein, and cytoplasmic markers such as annexin I, actin, tubulin, and other cytoplasmic proteins [12] are not present in NETs.

In the myeloid system, MPO plays a crucial role as a significant inflammatory factor [13]. Following an episode of acute cerebral ischaemia, the BBB is compromised, and a significant number of neutrophils are allowed to enter and attack the central nervous system. The considerable increase in the neutrophil population is linked to a notable increase in MPO synthesis [14, 15]. MPO has been implicated in the pathogenesis of stroke because it induces arterial wall damage through oxidation and its indirect impact on the integrity and functioning of blood vessels. This process contributes to the accumulation of low-density lipoprotein within a lipid-rich core and encourages the development of atherosclerotic plaques through the action of foamy macrophages [16]. During the process of NET formation, neutrophil elastase (NE) has multiple functions. Initially, NE translocates towards the nuclear envelope, facilitating the degradation of the nuclear membrane and the cleavage of histones. Furthermore, NE splits gasdermin D (GSDMD), thereby activating it and inducing perforations in the plasma membrane, thereby facilitating the extrusion of nuclear DNA [17]. In addition, extracellular traps (ETs) are no longer exclusively limited to neutrophils. Additional immune cells, such as LETs, MCETs, EETs, and METs, have been shown to release extracellular DNA. It is important to recognize and comprehend the arrangement and operation of these various origins of ETs.

NETs are particularly essential for immobilizing and capturing bacteria, fungi, or viruses, thereby improving the efficacy of pathogen elimination. This concept is supported by the increased vulnerability to various pathogens observed in mice lacking the ability to produce NETs, highlighting the critical role of NETs in infection. Moreover, NETs may also be significantly involved in noninfectious diseases and conditions such as systemic lupus erythematosus (SLE), diabetes, rheumatoid arthritis (RA), vasculitis, atherosclerosis, cancer, thrombosis, wound healing, and trauma. In addition, NETs have the potential to inflict harm upon host tissue, foster the emergence of autoimmunity, and give rise to various maladaptive consequences, such as metastasis, thrombosis, and aberrant coagulation.

### NETosis

The hallmark of NETosis is the liberation of DNA structures resembling webs, adorned with histones and cytotoxic proteins, from activated neutrophils [18]. NETs are activated by cholesterol, microcrystals, certain cytokines, antibodies, and pharmacological stimuli, including PMA, potassium ionophores, and calcium [19]. The presence of several substances, such as c-Raf, NADPH oxidase, ROS, and MPO, is required for the formation of NETs [20]. Following a sequence of reactions, the nuclear membrane disintegrates, chromatin decreases, and protein modifications occur, ultimately leading to the extrusion of NETs from the cell.

**Fig. 1:**
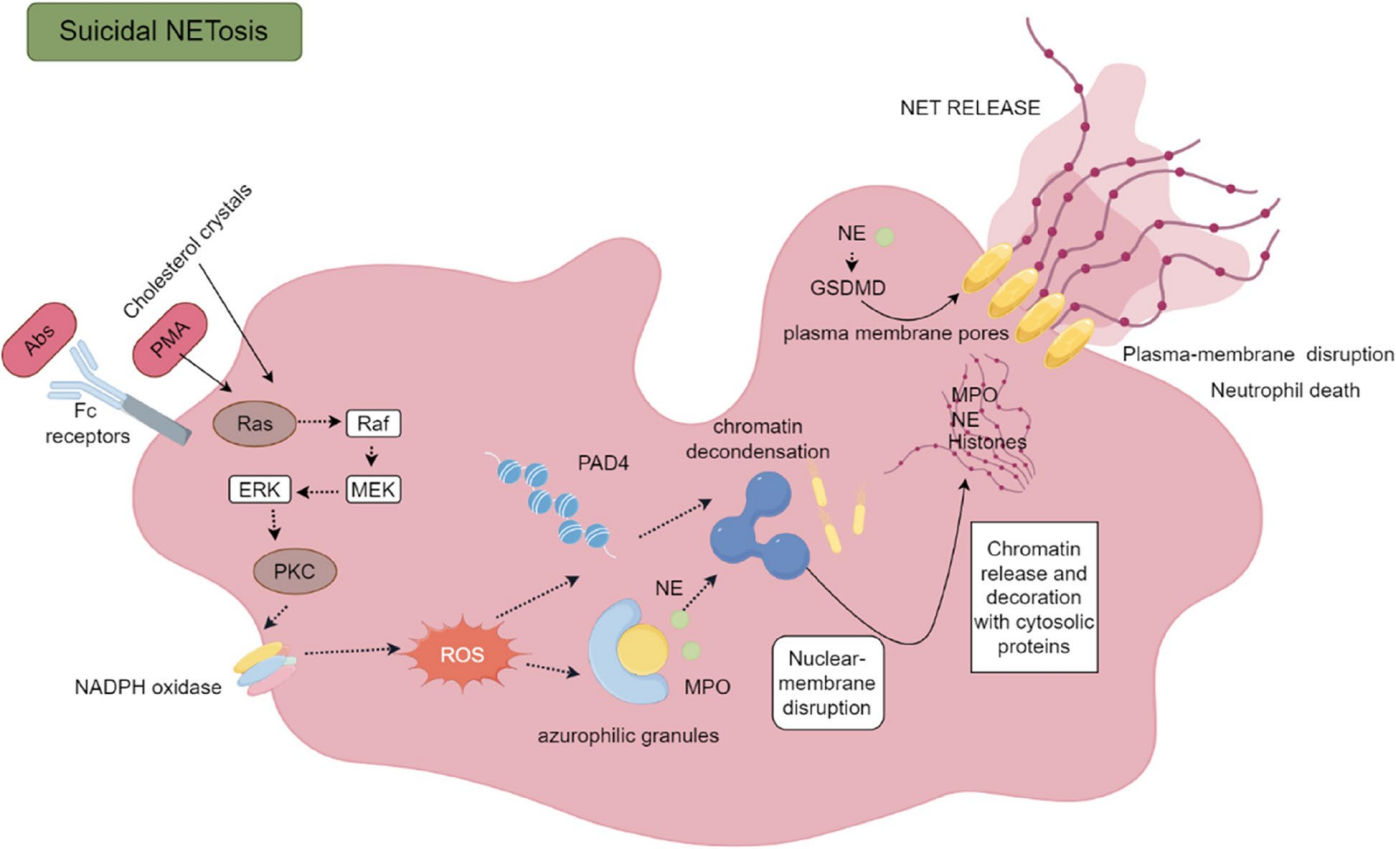
Suicidal NETosis: The activation of neutrophils by various stimuli, such as Abs, PMA, or cholesterol crystals, initiates a cascade of events after hours. At first, Ras is triggered, which is then followed by the activation of PKC via the Raf-MEK-ERK pathway. As a result, PKC triggers the generation of ROS through NADPH oxidase. The presence of ROS initiates the breakdown of azurophilic granules, resulting in the release of NE and MPO. Furthermore, the activation of PAD4 plays a role in chromatin decondensation. After the nuclear membrane is disrupted, the chromatin is released into the cytosol, where it undergoes changes with the help of granular and cytoplasmic proteins. Ultimately, GSDMD disturbs the cell membrane, resulting in the release of NETs into the extracellular space and the subsequent death of neutrophils

**Fig. 2:**
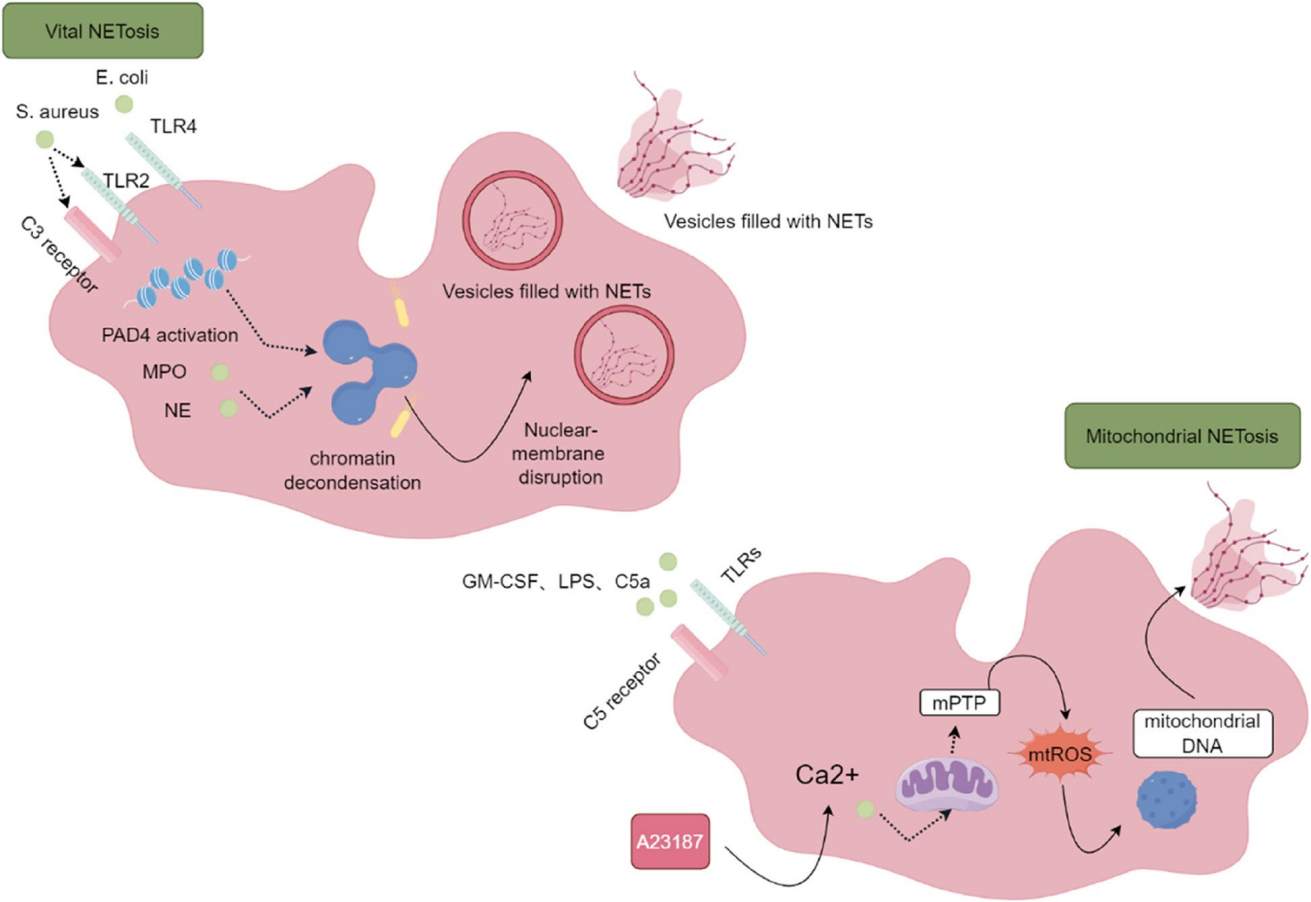
Vital NETosis: Activation of PAD4 by *Staphylococcus aureus* and LPS occurs within 5-60 minutes after neutrophils are stimulated with the complement proteins TLR2, TLR4, and C3 and after the transfer of NE to the nucleus, which induces chromatin decondensation and subsequent destruction of the nuclear membrane. Notably, this process does not require the involvement of NADPH oxidase. Finally, protein-modified chromatin is expelled through vesicles, allowing neutrophils to survive and maintain their functionality. Mitochondrial NETosis: After neutrophils are stimulated by GM-CSF, LPS, and C5a, a subsequent increase in the Ca2+ concentration in mitochondria is induced by A23187, leading to the activation of the opening of the mPTP and the generation of mtROS. Following a process akin to suicidal NETosis, mitochondrial DNA releases NETs

Recent extensive studies have been carried out to clarify the mechanisms underlying NETosis, resulting in the identification of three distinct mechanisms of NET formation. The initial form of NETosis, known as suicidal NETosis, is dependent on NADPH oxidase and is accompanied by neutrophil death. Suicidal NETosis is commonly referred to as the first type of NETosis and typically occurs within 2 to 4 hours after neutrophil activation. The precise process involves the combination of PMA or bacteria with receptors, which leads to the activation of Ras and subsequent activation of PKC via the Raf-MEK-ERK pathway. NADPH oxidase triggers the production of ROS in response to PKC stimulation [21]. The generation of ROS by NADPH oxidase subsequently leads to the activation of protein-arginine deiminase 4 (PAD4), an enzyme responsible for converting arginine to citrulline on histones. This enzymatic activity leads to chromatin decondensation within the nucleus of neutrophils. Additionally, NE and MPO are discharged from a specific set of neutrophil granules, known as azurophilic granules, into both the cytosol [22] and the nucleus, facilitating additional unravelling of chromatin [23]. Consequently, the nuclear membrane undergoes rupture, leading to the release of chromatin into the cytosol, where it undergoes additional modification through its association with granular and cytosolic proteins. Subsequently, nuclear DNA and antimicrobial compounds are released into the surrounding environment, and cell death is aided by the opening of the plasma membrane [21], potentially triggered by GSDMD activation. Furthermore, GSDMD may induce neutrophilysis, although the precise underlying mechanism remains unknown (Fig. 1). Vital NETosis, the secondary manifestation of NETosis, is characterized by the absence of neutrophil death and independence from NADPH oxidase. This process typically occurs within a time span ranging from 5 to 60 minutes after neutrophil activation. The initiation of this particular form of NETosis is triggered by the activation of C3 complement protein, Toll-like receptor 2 (TLR2), and Toll-like receptor 4 (TLR4) upon the stimulation of neutrophils with LPS and *Staphylococcus aureus* [24] .Vital NETosis can manifest autonomously from oxidants and is devoid of neutrophil lysis or plasma membrane rupture. Nevertheless, vesicle budding and the separation of the outer and inner nuclear membranes are observed. Moreover, both the vesicles and isolated membranes are replete with nuclear DNA. After the nucleus is expelled by vesicles, these neutrophils retain their capacity to engulf pathogens, and the depletion of DNA does not compromise their longevity. Mitochondrial NETosis, the third type, typically occurs within 15 minutes of the activation of neutrophils. The stimulation of neutrophils by complement factor 5a (C5a), LPS or granulocyte/macrophage colony-stimulating factor (GM-CSF) leads to an increase in Ca2+ levels in mitochondria, leading to the activation of the mitochondrial permeability transition pore (mPTP) and the generation of mitochondrial reactive oxygen species (mtROS). The formation of NETs is facilitated by the release of mitochondrial DNA, while nuclear DNA remains unaffected. Neutrophil death is not observed during this process [21, 25, 26]. However, the mechanisms underlying NET formation are still a subject of debate, and further investigation is needed (Fig. 2).

NETosis plays a crucial role in immune defence responses through these three distinct mechanisms. Several studies have demonstrated that numerous bacteria can directly adhere to extracellular DNA under controlled laboratory conditions. Consequently, it is hypothesized that the main function of NETs is to trap bacteria through DNA binding, suggesting the possibility of alternative mechanisms for bacterial capture. Furthermore, it is noteworthy that within NETs, multiple constituents such as MPO and NE, exhibit the ability to directly eliminate pathogens. Additionally, histone proteins exhibit potent antimicrobial properties [27], and DNA itself can impede microbial growth.

NETosis, a type of programmed cell death, may be interconnected with other cell death pathways. NETs can stimulate the production of interleukin-1β (IL-1β), which is recognized for its vital involvement in cell pyroptosis. Furthermore, NETs generated through GSDMD-dependent pyroptosis can further increase cell pyroptosis through a positive feedback loop, suggesting that NETosis may be involved in the induction of pyroptosis. Additionally, the involvement of caspase 8 in both apoptosis and necrotic apoptosis implies that if NETs influence caspase 8 activity, NETosis may also contribute to apoptosis and necrotic apoptosis. In general, GSDMD and caspase-8 could play key roles in NETosis, necroptosis, pyroptosis, and apoptosis [21]. Additional research is needed to validate this hypothesis (Fig. 5).

## The role of NETs in stroke pathology

### NETs affect inflammation and thrombosis

Progress in the investigation of the function of neutrophils in inflammation and thrombosis has resulted in a growing body of evidence that emphasizes the vital role of NET formation in these biological phenomena [28]. Numerous researchers have identified the involvement of NETs in thrombosis, facilitated by prethrombotic mediators such as tissue factor (TF), factor XII (FXII), microparticles (MPs), von Willebrand factor (vWf), and fibrinogen. Additionally, a colocalization network comprising fibrinogen, extracellular DNA, and vWF is observed within a thrombus, which has implications for various inflammatory and thrombotic diseases, including AIS and deep vein thrombosis (DVT) [29]. Local intraluminal exposure to elevated levels of TF generated by NETs within thrombi is crucial for the initiation and propagation of venous and arterial thrombi [28]. Some findings suggest that FXIIa contributes to the prethrombotic potential of NETs [30]. Additionally, the activation of neutrophils also results in the generation of a substantial quantity of neutrophil-derived MPs, which have a high abundance of phosphatidylserine (PS) on their external surface. This PS-rich environment can attract procoagulant factors. MP can bind to NETs via a histone-phosphatidylserine interaction. This interaction leads to the induction of thrombin production through the intrinsic pathway of blood clotting by the NET-MP complex [31]. Additionally, the aggregation of NET-MP complexes serves as a potent stimulant for neutrophil recruitment in vivo. These findings suggest that the presence of MPs attached to NETs is crucial for promoting neutrophil adhesion and exosmosis within the microvascular system. Furthermore, the proinflammatory effects of the NET-MP complex are triggered by the high mobility group box 1 (HMGB1) expressed on MPs, which activates the TLR2 and TLR4 signalling pathways [32]. HMGB1 influences the formation of NETs through its interaction with TLR2, TLR4, and receptor for advanced glycation end products (RAGE), and this process is dependent on NADPH oxidase. The inhibition of HMGB1 may serve to mitigate the inflammatory damage caused by ANCA-induced NET formation [33].

### NETs in stroke thrombi

Traditionally, it has been widely accepted that blood clots mainly comprise platelets, fibrin, and entrapped red blood cells. However, leukocytes also play a crucial role in thrombosis. When stroke thrombi were analysed, H&E staining indicated a notable occurrence of leukocytes, which could be observed as cells with nuclei in every thrombus sample. Elodie et al. documented the existence of NETs in clots, albeit in varying quantities [34]. Their research demonstrated that some thrombi exhibited fewer neutrophils, while others displayed more of these cells. Notably, no discrepancies in neutrophil numbers were observed among thrombi with varying causes. Surprisingly, there was a significant increase in neutrophil numbers in older thrombi compared to those in fresh thrombi, and there was a greater prevalence of neutrophils in individuals with cardioembolic thrombi than in individuals with noncardioembolic thrombi. Activated neutrophils can activate and inflict damage upon the endothelial cells lining the vascular lumen [35–37]. Additionally, activated endothelial cells can initiate the release of NETs [38, 39]. This process initiates the degradation of these cells and establishes a detrimental feedback loop between neutrophils and endothelial cells. Ultimately, this loop can culminate in the formation of thrombi.

Platelet-rich regions in stroke clots exhibit a complex composition, encompassing diverse scaffolds such as fibrin, vWF, and DNA. The dense fibrin structure aligns with vWF and accommodates platelets [31]. In the context of an experimental mouse model investigating cerebral I/R injury in ischaemic stroke, a notable decrease in NET formation following ischaemia was detected in VWF-deficient mice compared to their wild-type counterparts. Consequently, targeting VWF is promising as a compelling avenue for the advancement of novel therapeutic approaches for the management of ischaemic stroke [40]. Mechanistically, NETs function as a structure for the attachment of platelets and red blood cells, as well as the aggregation of procoagulant proteins. Interactions between NETs and other molecules can occur through various mechanisms. For instance, the attachment of vWF, fibronectin, or fibrinogen to NETs can facilitate the binding of platelets. Additionally, platelets can directly interact with DNA histones within NETs. Furthermore, the strength and durability of blood clots formed by NETs and fibrin as thrombotic structures are remarkably robust. In fact, the formation of clots when activated neutrophils are present can be averted solely through concurrent administration of tissue plasminogen activator (tPA) and DNase [41]. In certain individuals experiencing myocardial infarction, the exposure of NETs to TF can stimulate the generation of thrombin. Subsequently, thrombin activates quiescent platelets via PAR cell signalling, thereby promoting the formation of NETs by neutrophils. Alterations in thrombin levels have been shown to impact the production of NETs, suggesting the potential involvement of thrombin in this biological process [42]. The novel phthalide CD21, a neuroprotective agent, has the potential to modulate the platelet-NET-thrombin axis and mitigate ischaemic brain injury through the activation of AMPK. CD21 administration to stroke mice has been shown to improve regional cerebral blood flow, neurobehavioural deficits, and infarct volume [43]. Notably, CD21 has been shown to significantly reduce the levels of NET components, PDA4, and NET-related inflammatory mediators [44]. However, the effects of CD21 were effectively counteracted by pretreatment with AMPK [45]. The release of neutrophils and cytoplasmic components involved in NETs is crucial for maintaining IL-17-driven neutropenia and promoting NET formation [46]. In addition, NETs induce the synthesis of IL-1β in macrophages. Subsequently, IL-1β stimulates Th17 lymphocytes, resulting in the secretion of IL-17, which subsequently increases the influx of immune cells into atherosclerotic plaques. Additionally, it has been observed in thrombectomy samples that NETs coated with IL-17 increase platelet aggregation, thereby promoting thrombosis [47]. The involvement of NETs in the pathogenesis of stroke thrombosis has been demonstrated in clinical cases. Carotid stenosis, accounting for 15% of all strokes, has been identified as a causative factor [48]. Differential gene expression analysis revealed distinct patterns between plaques extracted from patients with carotid stenosis and control specimens. Interestingly, enrichment analysis did not reveal significant differences in PAD4 expression between the two groups, and the presence of NETs was observed in only one cDNA microarray dataset. However, it is important to note that these findings do not definitively exclude the possibility of a relationship between NETs and atherothrombotic stroke. Additional research is need on the differential gene expression between endothelial cells and atherosclerosis [49].

Additional aetiologies of stroke include atherothrombosis (AT), cardiogenic embolism (CE), and cryptogenic stroke. AT thrombi exhibit a greater abundance of lymphocytes, whereas cryptogenic thrombi display a reduced presence of macrophages. Remarkably, CE clots exhibit increased neutrophil and extracellular reticular NET expression [50]. NETs play a significant role in a diverse array of illnesses. Consequently, further investigation into the involvement of NETs in stroke thrombosis and various diseases is imperative.

### NETs are markers of stroke

Leucocytosis, characterized by a predominance of neutrophils, is common in the majority of patients with AIS, regardless of the underlying cause of the stroke. This phenomenon holds potential as a noteworthy biomarker. In individuals with AIS, the normal functioning of neutrophils is disrupted. Research has shown that neutrophils exhibit an increased propensity to form NETs in the context of AIS. Moreover, the resolution of NETs was more challenging even after the administration of DNase-I in AIS patients than in healthy individuals. Consequently, it is hypothesized that NETs not only serve as an immune response to ischaemic events but also contribute to the induction of thrombosis [51].

Several markers, including MPO-DNA, citH3, cfDNA, nucleosomes, DNase-I, and circulating CitH3, have been identified as potential indicators of the level of NETs and can be utilized as biomarkers for predicting ischaemia in peripheral artery disease and coronary artery disease [52, 53]. Research has demonstrated that the levels of both neutrophil and NET markers are similarly high in the lysate supernatants of thrombi retrieved after mechanical thrombectomy. Furthermore, the levels of NET markers and the absolute granulocyte count in the peripheral blood of patients with acute stroke were found to be significantly elevated [6, 51, 54]. Patients with COVID-19 who experienced a stroke exhibited more neutrophils in their peripheral blood, along with significantly greater concentrations of dsDNA associated with the NET marker, than did control participants. Additionally, a significant correlation was observed between the NET content present in AIS thrombi and the NET content found in peripheral blood [55, 56]. Moreover, a parabolic relationship was identified between systemic inflammatory markers and NET components, while a positive correlation was observed with patient age [57]. Furthermore, it was discovered that the subgroup of patients with diabetes and acute hyperglycaemia had substantially elevated levels of NETs compared to individuals without these conditions [58]. Additionally, the correlation between the cumulative count of serum neutrophils and the concentrations of plasma NET markers in individuals experiencing acute stroke is linked to the severity of the stroke, as indicated by the NHISS score and infarct size, and is associated with an unfavourable prognosis [59].

Evidence suggests that increased levels of CitH3 are associated with more extensive white matter lesions (WMLs) in patients with ischaemic stroke. These WMLs are characterized by anomalous white matter observations on MRI of the brain in individuals with ischaemic stroke, particularly those of advanced age, and are influenced by various vascular risk factors [60, 61]. The presence of WMLs has been found to be correlated with unfavourable functional outcomes, elevated mortality rates, an increased likelihood of bleeding after thrombolysis, and long-term recurrence following ischaemic stroke [62–66].

Furthermore, no association between stroke severity at admission and immune thrombotic biomarkers, including D-dimer, platelet factor 4, calprotectin, the MPO-DNA complex, or DNase activity, has been observed. However, markers indicative of NET formation, such as H3cit and MPO-DNA levels, exhibit a positive correlation with stroke outcomes [67].

### NETs impair brain function after ischaemic stroke

#### NETs promote vascular injury and neurological deficits

NETosis has been demonstrated to augment inflammation and subsequent cerebral injury. Despite the ineffectiveness of inhibiting NETosis in reducing infarct volume in animal models, a clear amelioration of delayed inflammation and vascular damage was observed [68].

New findings indicate that the development of new blood vessels after brain injury is of utmost importance in facilitating the innate healing process [69, 70]. Furthermore, impeding the growth of these blood vessels exacerbates the consequences of cerebral ischaemia [71]. Consequently, the acceleration of revascularization has substantial therapeutic potential for treating diverse central nervous system disorders. Previous investigations have underscored the significance of the cortical regions surrounding the infarct in fostering functional recovery in animal stroke models [72, 73]. Increased survival in stroke patients is also associated with increased vascularization in these regions [74]. Nevertheless, these recently developed vessels are currently porous and undergoing maturation [75]. The opening of the BBB results in an increase in the leakage of immune cells and toxic proteins derived from blood [76, 77]. Consequently, it is imperative to maintain the stability of vessels and restore the impaired BBB to ensure a stable brain microenvironment.

A comparison between ischaemic model mice and control mice revealed that neutrophil accumulation in the brain was observed at all stages in the ischaemic model mice. Furthermore, increased production of NETs, which release various cytotoxic proteins and ROS, thereby inducing endothelial damage, was observed. However, when neutrophils from stroke mice were depleted of anti-LY6G antibodies, damage to the BBB was alleviated, and there was an increase in the formation of new blood vessels and vascular perfusion [78]. These findings indicate that neutrophils play a crucial role in the formation and maintenance of new blood vessels following stroke. This process is dependent on the activity of PAD4, an enzyme involved in chromatin decompensation [79, 80]. For example, mice overexpressing PAD4 showed a decrease in vascular branches, microvascular length, and perfused cortical microvascular length. Additionally, these mice displayed more severe neurological impairments, as assessed by the beam walking test and forelimb force test.

A separate study revealed that the presence of NETs within ischaemic thrombi can exacerbate the extent of brain damage caused by ischaemic stroke. The researchers conducted postmortem examinations on patients who died from ischaemic stroke and observed increased levels of NETs and platelets, indicating an upregulation of their formation. HMGB1, a nonhistone protein, plays a role in the condensation and packaging of intranuclear DNA through its nonspecific interactions with DNA. The involvement of HMGB1 in various cellular processes, such as transcription, replication, repair, and recombination, has been shown to be attributed to its interaction with DNA. HMGB1 is also a damage-associated molecular pattern (DAMP) that has recently been proposed to be a potential inducer of NETosis in noninfectious diseases [68]. Jin et al. reported that the production of NETs is facilitated by activated platelets containing HMGB1 [81]. Additionally, a team observed a similar increase in NETs and neutrophils in a mouse model of transient middle cerebral artery occlusion (tMCAO) and noted a reduction in HMGB1 and NETs, an alleviation of brain damage and improved neurological function after platelet depletion; the opposite results were observed when recombinant HMGB1 was administered. The release of HMGB1 induces NET formation in ischaemic stroke through platelet activation, leading to a worsened prognosis for stroke patients [67, 82]. The expression of HMGB1 is increased on platelet microbubbles (PMBs) and stimulates the formation of NETs in the AIS. These NETs contribute to increased procoagulant activity (PCA) by activating tissue factor and platelets, thereby exacerbating thrombosis and brain injury in AIS patients [83].

In the context of the signalling pathway associated with brain injury induced by NETs, researchers have observed a substantial increase in cortical levels of interferon-beta (IFN-β) and stimulator of interferon genes (STING) subsequent to a stroke event. Additionally, downstream of the STING signalling pathway, the levels of phosphorylated TANK-binding kinase 1 (pTBK1) and interferon regulatory factor 3 (IRF3) were found to be elevated. Neutralization of IFN receptors, depletion of neutrophils or treatment with a PAD4 (Cl-amidine) inhibitor resulted in a reduction in the STING signalling induced by stroke. These results indicate that stimulation of the STING pathway through NETs and suppression of the type I IFN reaction may alleviate damage caused by stroke and improve stroke outcomes [78].

In mammals, the genes PKLR and PKM encode four distinct isoforms of pyruvate kinase (PK), namely, PKR, PKL, PKM1, and pyruvate kinase muscle 2 (PKM2). PKR is predominantly found in red blood cells; PKL is primarily expressed in the liver and kidney; and PKM1 is mainly present in mature adult tissues that require a substantial amount of adenosine triphosphate, such as brain, heart, and muscle tissues. In contrast, PKM2 is detected in various tissues, including lung and spleen tissues, and all cancer cell lines [84]. Research has highlighted the importance of the glycolytic enzyme PKM2 in regulating aerobic glycolysis. Additionally, PKM2 has been found to contribute to the activation of proinflammatory mediators such as IL-1β and IL-6, thus playing dual roles [85–87]. After cerebral ischaemia and reperfusion, mice without PKM2 displayed a decrease in the levels of inflammatory cytokines (IL-1β, TNF-α, and IL-6) and a decrease in proinflammatory gene expression (elastase, MPO, IL-1β, and HIF1α) within neutrophils, along with a decrease in NET production. Consequently, decreases in the size of the cerebral ischaemic area and the inflammatory response were observed, ultimately resulting in improved neurological outcomes and motor function. This effect can also be achieved by using the small molecule ML265 to inhibit nuclear PKM2 translocation. Additionally, PKM2 plays a role in regulating immune cells, including activating T cells and augmenting antitumour and proinflammatory functions [88–90].

**Fig. 3:**
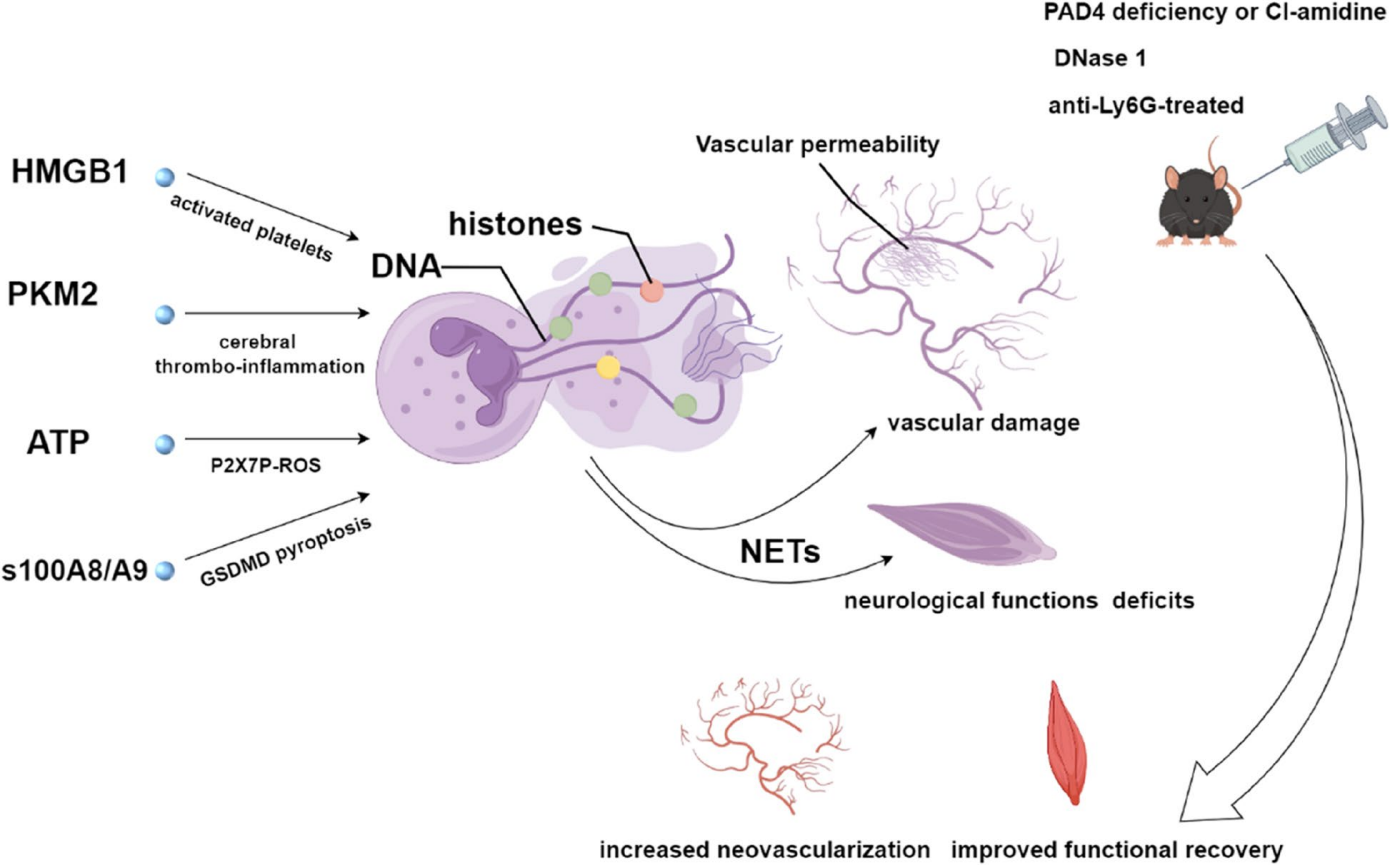
Elevated levels of NETs were observed in both brain tissue and peripheral blood samples obtained from tMCAO model animals and stroke patients. The formation of NETs is believed to be influenced by various factors, including HMGB1, PKM2, s100A8/A9, and ATP. HMGB1, a nonhistone protein, is induced by platelet activation and interacts with DNA to facilitate the release of NETs. PKM2, a subtype of pyruvate kinase, promotes the expression of neutrophil inflammatory factors and increases the production of NETs. ATP serves as an activator of P2X7R in neutrophils, thereby initiating NETosis through the generation of ROS. Additionally, s100A8/A9 selectively targets Toll-like receptors, leading to platelet pyroptosis, which subsequently facilitates the formation of NETs. Consequently, the integrity of the blood–brain barrier is compromised, neovascularization is impeded, and vascular damage ensues, accompanied by nerve impairment resulting in diminished limb functionality. Nonetheless, the detrimental consequences of these lesions can be alleviated by inhibiting NETs with Ly6G, PAD4 inhibitors, and DNase1

The accumulation of ATP is sufficient to activate P2X7R on neutrophils, leading to the induction of NETosis in a manner dependent on P2X7R, Ca2+, and ROS. Additionally, the induction of NETosis by ATP is similarly facilitated by the prototypic P2X7R agonist BzATP, which suggests the establishment of a detrimental cycle driven by ATP that exacerbates the inflammatory response following permanent MCAO [91] (Fig. 3).

#### NETs influence inflammasome activity and pyroptosis in brain injury

When examining the impact of ATP on the production of NETs, it is crucial to acknowledge that ATP can indirectly induce NETosis by activating the P2x7R-mediated NLRP3 inflammasome. This activation subsequently results in cell death and the subsequent release of NETs. Consequently, we further investigated the mechanism through which NETs influence injury, specifically by examining the role of pyroptotic cell death and the NLRP3 inflammasome. The NLRP3 inflammasome acts as a polymeric protein platform in the cytoplasm, initiating the activation of the protease procaspase 1 (CASP1). Inflammasome activation is essential for the proteolytic activation of proinflammatory cytokines, specifically IL-1β and IL-18, and the initiation of the form of programmed cell death called pyroptosis. The NLRP3 inflammasome functions as a cytoplasmic polymeric protein platform that triggers the activation of the protease. Inflammasome activation plays a crucial role in the proteolytic activation of proinflammatory cytokines, namely, IL-1β and IL-18, as well as the induction of pyroptosis. In addition to macrophages, dendritic cells (DCs), and monocytes, neutrophils can also form inflammasomes when exposed to different stimuli, which has important functional implications. Regarding the inflammatory bodies of neutrophils, human neutrophils secrete IL-1β in vitro in a manner dependent on NLRP3 [92, 93]. However, compared to macrophages, neutrophils secrete IL-1β in smaller amounts and at a higher rate in terms of kinetics [94]. The dominance of TNF-α in initiating the neutrophil inflammasome compared to its contribution to macrophages has been observed in previous studies [95–97]. Furthermore, the NLRP3 inflammasome also serves as a mediator of NETosis and thrombosis, highlighting the importance of NLRP3 inhibition as a novel therapeutic approach for thrombotic diseases [98]. Inhibiting the PKM2-eif2AK2 pathway pharmacologically or using conditional knockout of PKM2 in bone marrow cells protected mice from the detrimental effects of NLRP3 and AIM2 inflammasome activation [99]. Neutrophils activate the NLRC4 inflammasome in response to *Pseudomonas aeruginosa* and are capable of inducing pyroptosis during "incomplete NETosis" [97]. In relation to the phenomenon of platelet pyroptosis, investigations conducted on mice lacking platelet-specific GSDMD revealed that GSDMD serves as a trigger for platelet pyroptosis in sepsis induced by CLP. This process is stimulated by elevated levels of S9A4/A4, which specifically target TLR8. Additionally, the release of platelet-derived oxidative mitochondrial DNA (ox-mtDNA) may facilitate the generation of NETs, thereby promoting platelet pyroptosis through the release of S100A8/A9. Consequently, a positive feedback loop is established, leading to an excessive release of inflammatory cytokines [100] (Fig. 3). A set of experiments involving bone marrow transplantation, neutrophil depletion, and RNA sequencing has demonstrated the crucial role of neutrophil-specific GSDMD in the generation and provision of neutrophils within the bone marrow. The absence or pharmacological inhibition of GSDMD leads to a decrease in neuronal death and infarct volume while also improving neural function in mice. Additionally, GSDMD knockout results in reduced inflammation and neutrophil numbers in the brain, blood, and bone marrow [101].

### NETs promote tPA-induced brain haemorrhage

#### t-PA thrombolytic therapy for ischaemic stroke

Ischaemic stroke is characterized by the occlusion of one or multiple arteries in the brain due to the presence of a blood clot. This occlusive thrombus obstructs blood flow, resulting in irreversible damage to the corresponding cerebral tissue. The primary approach in the current treatment of ischaemic stroke involves promptly removing blood clots to restore blood flow through the affected vessel as early as possible. This approach aims to reduce tissue damage and improve the patient’s prognosis [102]. Currently, there are two U.S. Food and Drug Administration (FDA)-approved therapeutic approaches for the removal of thrombi: endovascular thrombectomy, which involves the mechanical eradication of the thrombus, and pharmacological thrombolysis employing tissue plasminogen activator (t-PA), a compound that facilitates fibrin degradation within the obstructive thrombus.

Following the administration of r-tPA, a notable proportion of stroke patients, ranging from approximately 17% to 34%, experience early reocclusion in the arteries and a less favourable long-term outcome. A precise explanation for this phenomenon, known as "t-PA resistance", is lacking; however, it seems that the composition of the thrombus significantly influences t-PA resistance occurrence [103]. Despite being responsible for fibrin degradation and having broad specificity, the enzyme rt-PA has not been proven to be an effective thrombolytic drug in more than 50% of patients. The success of intravenous thrombolysis (IVT) in the treatment of AIS depends not only on achieving arterial recanalization but also on the promptness of the procedure. It is plausible that the clinical failure of IVT may be attributed to the inadequate speed or efficacy of fibrinolysis itself, resulting in delayed recanalization. Studies suggest that the challenges in determining the efficacy of IVT extend beyond the delivery of rt-PA to the thrombus, indicating the presence of resistance [34, 104, 105]. Subsequent analysis revealed that the shell and platelet-rich regions of AIS thrombi play a significant role in causing resistance to rt-PA treatment [106]. Additionally, the presence of VWF in AIS thrombi was found to be unaffected by rt-PA administration [105]. Recent investigations focusing on neutrophils have demonstrated that NETs can serve as an antifibrinolytic framework, enabling clot formation even in the presence of rt-PA-induced fibrin degradation [41]. DNase 1, on the other hand, can expedite the rt-PA-mediated dissolution of arterial thrombi and may also serve as an adjunctive therapy for thrombolysis [107]. Notably, the lytic effect of DNase 1 on human AIS thrombi was not found to be significant in the absence of rt-PA [108]. Our recent findings demonstrated that the thrombolytic advantage of extracellular DNA degradation in human AIS thrombi is derived from the augmentation of rt-PA-mediated fibrinolysis [104]. Neutrophil elastase-mediated degradation of tissue factor pathway inhibitors and platelet regulatory proteins may occur through NETs in platelets. In contrast to DNase 1, the VWF-targeting agent possesses unique thrombolytic activity in mice, independent of rt-PA administration [109–111].

#### NETs play a role in the development of cerebrovascular complications associated with t-PA treatment

Despite its efficacy, tPA treatment is associated with an increased risk of intracerebral bleeding [112]. Nonetheless, a comprehensive understanding of the factors contributing to this bleeding remains incomplete.

It is currently acknowledged, t-PA can activate the brain endothelium, resulting in the deterioration of vascular integrity and the sudden disruption of the BBB [113, 114]. Neutrophils entering brain tissue from small blood vessels are crucial for the breakdown of the blood‒brain barrier following ischaemic stroke [115]. Notably, tPA was found to facilitate the enlistment of neutrophils to ischaemic tissue [116, 117]. Upon activation, neutrophils can release NETs [41, 118].

There is a significant link between neuroinflammation and the incidence and progression of bleeding following t-PA thrombolysis. This association encompasses the activation of glial cells within the brain, the infiltration of peripheral inflammatory cells, and the release of inflammatory factors. The effects of regulatory T cells (Tregs) were examined. These Tregs make up approximately 10-4% of CD5+ T cells in circulation [119], and their administration after regulatory T-cell infusion significantly mitigated tPA-induced cerebral haemorrhage in both suture and embolization models [120]. Further mechanistic investigation revealed that in thrombolysis models of ischaemia/reperfusion, Tregs contribute to BBB protection through one or more metalloproteinase-9 (MMP9)-independent mechanisms. Additionally, tPA treatment was found to increase CCL2 expression in both ischaemic brain and in vitro BBB models. However, Treg treatment significantly inhibited this induction of CCL2. Consequently, inhibiting CCL2 is anticipated to decrease neutrophil infiltration [121], thereby reducing the accumulation of factors that disrupt the BBB, such as MMP9 [120]. Furthermore, the administration of rtPA at 4 hours following ischemia resulted in the upregulation of NLRP3 expression in neurons, microglia, and endothelial cells, which subsequently led to the degradation of components of the BBB and the occurrence of post-thrombolytic bleeding. However, the knockdown of NLRP3 significantly mitigated the expression of NLRP3, the destruction of the BBB, and the development of haemorrhagic transformation (HT). Additionally, NLRP3 knockdown has been shown to improve nerve function and reduce the recruitment of neutrophils [122]. Following tPA thrombolytic therapy for ischaemic stroke, a novel pathway of microglial activation has been identified. This pathway occurs when tPA interacts with low-density lipoprotein receptor-related protein 1 (LRP-1), leading to microglial activation and harmful inflammatory reactions. Indeed, in vitro investigations have demonstrated that the binding of tPA to LRP-1 increases the production of matrix MMP-9 [123, 124] and initiates the activation of nuclear factor kappa B (NF-κB), resulting in an increase in the expression of inducible nitric oxide synthase (iNOS) [125, 126]. Furthermore, the use of tPA led to an increase in the number of microglia/macrophages, polarization towards the M1 phenotype, an increase in the presence of macrophage inflammatory protein-1 alpha (MIP-1α) in neurons and capillaries, and an increase in the expression of metalloproteinase-3 (MMP3) in rats 4.5 hours poststroke. Notably, these inflammatory and destructive responses were effectively attenuated by progesterone (PROG) treatment [127].

A study conducted by Wang and colleagues revealed that in a mouse model of AIS induced by MCAO, the administration of tPA resulted in a notable increase in the number of neutrophils in the brain, and the local levels of MPO and H3Cit, which are markers of NETs, were also increased. The production of cytotoxic proteases, such as elastase, histones, proinflammatory mediators, and MPO, by NETs can harm the vascular endothelium and increase vascular permeability [78, 128]. Additionally, NETs contribute to the heightened depletion of gap junction proteins, including occludin, zonula occludens-1 (ZO-1), vascular endothelial-cadherin (VE-cadherin), and claudin-5, resulting in increased ICH and the worsening of neurological impairments, as evidenced by behavioural testing. However, the detrimental effects of NETs can be alleviated by removing NETs using DNase I or inhibiting PAD4 to reduce NET formation.

The process of tPA-induced NET formation was found to be associated with the presence of LRP-1 in neutrophils and the subsequent increase in PAD4 expression. Both the upregulation of PAD4 and the formation of NETs were inhibited by the antagonist receptor-associated protein (RAP), which blocks LRP-1. This study provides evidence for the involvement of the tPA-neutrophil-LRP-1-PAD4 pathway in the development of tPA-induced intracerebral haemorrhage following ischaemic stroke. Here, LRP is a large endocytic receptor on macrophages. The accumulation of Aβ within microvessels worsens damage to the endothelium according to the findings of BBB studies [129, 130]. LRP-1 facilitates the removal of Aβ from brain tissue to vessels through the BBB, resulting in damage to the BBB and vascular dysfunction [131].

**Fig. 4:**
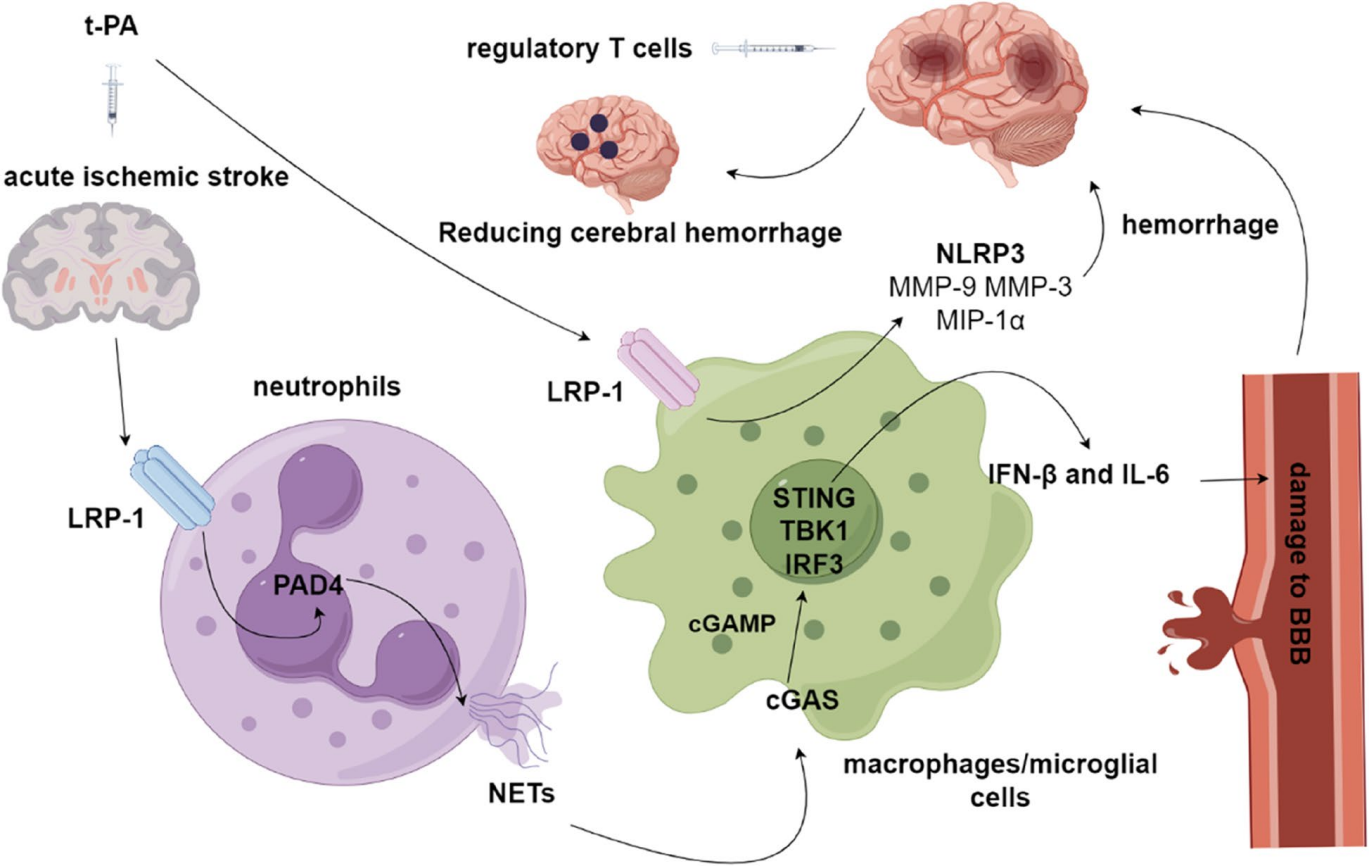
The administration of tPA led to a notable increase in the expression of LRP-1 and NETs within the ischaemic cortex of mice. Following tPA injection, it interacts with LRP-1 in various cells, thereby fostering the generation of NETs in neutrophils. Consequently, this process induces the expression of cGAS in microglia and stimulates the expression of IFN-B and IL-6 through STING, TBK1, and IRF-3. The infusion of regulatory T cells has been shown to ameliorate TPA-induced intracerebral haemorrhage by inhibiting the expression of NLRP3, MMP-9, MMP-3, and MIP-1α in microglia. These factors are known to contribute to an inflammatory response, blood‒brain barrier disruption, vascular endothelium damage, and subsequent cerebral haemorrhage

**Fig. 5:**
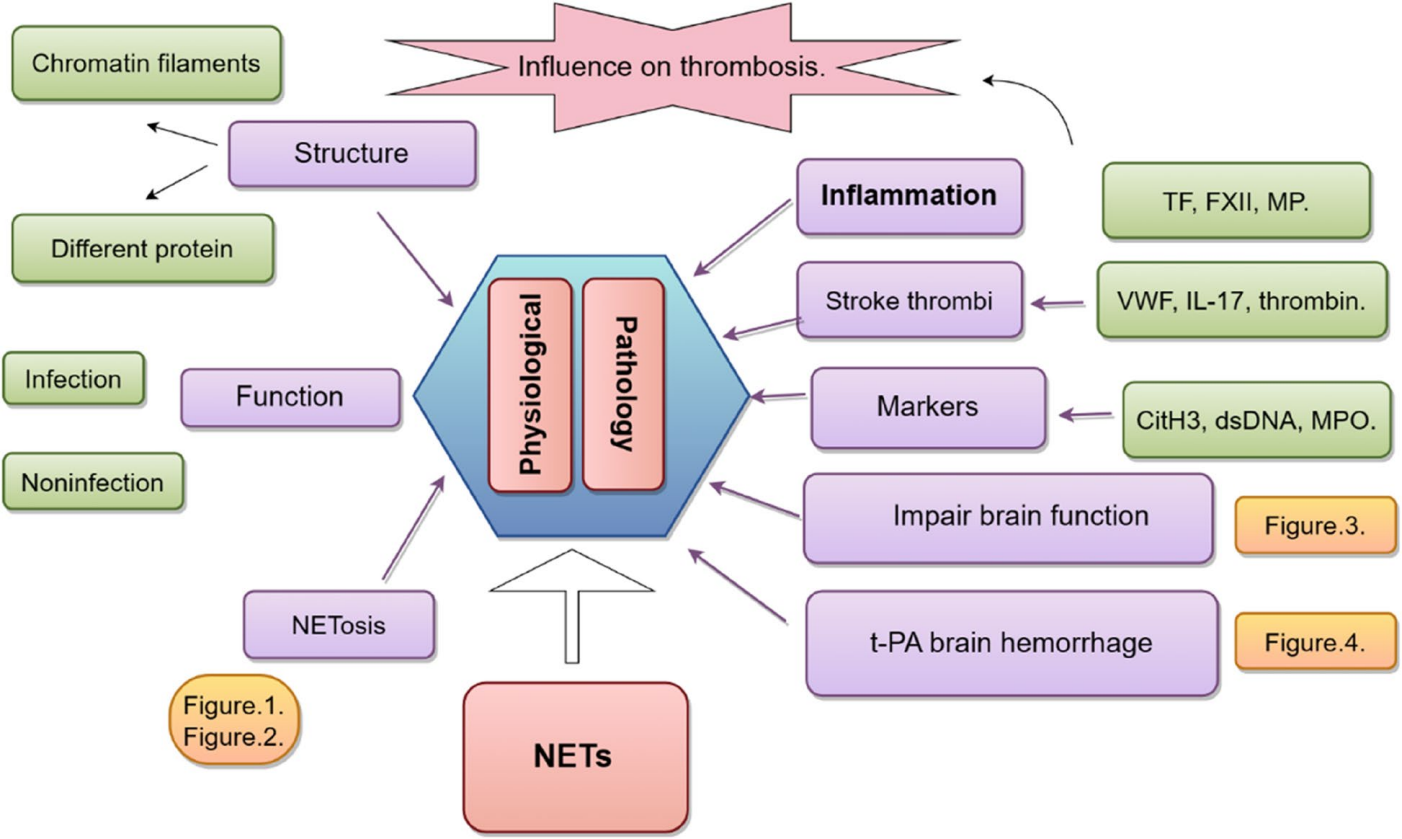
This figure briefly summarizes the contents of Sections 2 and 3

Researchers have identified a mechanism by which the release of genetic material by neutrophils induces the activation of the intracellular DNA receptor cyclic GMP-AMP synthase (cGAS) in macrophages and microglia in the ischaemic brain. The expression of cGAS in the ischaemic cortex of mice was consistently increased, and tPA further increased cGAS expression. The cGAS receptor facilitates the synthesis of cGAMP, which subsequently activates the STING/TBK1-IRF3 pathway, leading to the production of IL-6 and IFN-β. Treatment of ischemic mice with tPA resulted in a significant decrease in the activation of the cGAS-STING pathway and the production of IL-6 and IFN-β when DNase I and PAD4 deficiency were present (Figs. 4 and 5).

## Comorbidities of stroke affected by NETs

NETs induce the acquisition of a mesenchymal phenotype [132, 133]. The degradation of VE-cadherin, facilitated by neutrophil elastase, the primary protease within NETs, disrupts the integrity of intercellular junctions [134] The preservation of intercellular junction integrity is essential for maintaining the impermeability of the endothelial barrier. However, the degradation of VE-cadherin by elastases associated with NETs can lead to an increase in vascular permeability [134]. Haemorrhage may contribute to blood‒brain barrier disruption and cerebral haemorrhage following stroke. Pretreatment of NETs with sivelestat, a specific elastase inhibitor, effectively prevented the loss of VE cadherin expression induced by NETs [135, 136].

The immune alterations induced by stroke have been found to notably impair the essential processes involved in bacterial eradication, namely, oxidative burst and NETosis, consequently compromising the bacterial defence system. Specifically, stroke patients with stroke-related infections exhibit a more pronounced impairment in monocyte oxidative burst upon admission [137].

Obesity-induced hyperglycaemia is a significant risk factor for stroke [138]. In contrast to lean mice, obese mice experiencing stroke due to hyperglycaemia exhibit an increase in neutrophil α9 expression, leading to increased NET formation. Consequently, these mice experience a prolonged impairment in functional outcome for a duration of up to 4 weeks, accompanied by an exacerbation of infarction and a reduction in cerebral blood flow. This effect has the potential to exacerbate thromboinflammation following reperfusion [139].

Hypertension is associated with a greater stroke size, potentially attributable to a significant surge in poststroke inflammation. This phenomenon may be attributed to an elevated count and heightened activation status of circulating myeloid leukocytes, along with heightened levels of chemokines that attract leukocytes in hypertensive patients [140].

## NETs: Treatment for stroke

Following IVT and endovascular therapy (EVT), a significant proportion of treated patients do not achieve a favourable outcome [141, 142]. This lack of clinical effectiveness of reperfusion can be attributed to various factors, such as irreversible infarction, infarct evolution, haemorrhagic transformation, cerebral oedema, microvascular occlusion, and suboptimal postreperfusion care [143]. As a result, the Stroke Treatment Academic Industry Roundtable XII convened to address strategies for improving acute stroke outcomes [144]. Opportunities exist for improving poststroke functional outcomes through the reduction of treatment duration in eligible patients prior to reperfusion [145, 146], as well as the timely administration of neuroprotective agents following stroke onset [147]. The utilization of combination therapies to augment thrombolysis [148], address microcirculatory flow disorders [149], implement techniques and devices targeting nerves or neuromodulation [150, 151], and increase brain protection [152] all contribute to the optimization of IVT and EVT efficacy. Increased cerebral protection following reperfusion [153, 154], neuromodulation [155], and combined cell therapy [156] shows potential for increasing cerebral perfusion. Targeting NETs represents a promising reperfusion strategy with significant implications for the management of ischaemic stroke.

In both animal models of inflammatory diseases and laboratory settings, DNase I is commonly utilized as the principal enzyme for disrupting NETs. This approach is deemed safe due to its widespread acceptance and the limited occurrence of adverse reactions [157, 158]. DNase I breaks down NETs by specifically binding to double-stranded DNA (dsDNA) and extracellular nuclear proteins. This process effectively minimizes BBB damage, resulting in increased pericyte coverage on microvessels and the development of new functional vessels. These findings support the notion that NET formation contributes to vascular injury [103, 112]. Nonetheless, the primary concern regarding DNase I as a treatment is its comparatively brief duration of 3–4 hours and its swift deactivation by G-actin in the bloodstream. Histopathological examination of thrombi extracted from patients undergoing treatment for acute ischaemic stroke revealed that a longer time to recanalization was linked to higher levels of fibrin, H3Cit, and vWF within the thrombi [159]. This occurrence may be attributed to a heightened activation of coagulation within the thrombus as time progresses, consequently elevating the fibrin:red blood cell ratio of the thrombus. Additionally, the presence of neutrophil extracellular traps, which increase with the age of the thrombus, alters the structure of fibrin [34, 160]. Moreover, the duration of the recanalization procedure exhibits an inverse relationship with the infiltration of red blood cells and a direct relationship with the infiltration of fibrin within the thrombus [161]. These findings suggest that thrombi containing more red blood cells are more prone to successful recanalization [159, 162].

PAD4, a crucial enzyme, converts a positively charged arginine into a negatively charged citrulline residue, thereby citrullinating histones. This significant impact on cellular communication triggers the relaxation of chromatin structure, which aids in the removal of nuclear DNA [163]. Kang et al. observed a noteworthy increase in the expression of PAD4 in the peri-ischaemic cortex, which subsequently resulted in intensified vascular injury and diminished formation of new blood vessels due to the increased release of NETs. Consistently, the findings consistently demonstrated that the suppression of NET formation through either genetic knockout of PAD4 or the use of a pharmacological inhibitor resulted in a decrease in cerebrovascular integrity loss, an increase in neovascularization and capillary perfusion, and an increase in functional recovery. These findings indicated that increased NET formation by PAD4 caused impaired delayed vascular remodelling following stroke, as shown by these results. The involvement of NETs in the development of thromboinflammatory diseases has been established [164], with PAD4 and NOX playing significant roles in neutrophil-driven thromboinflammation. By targeting PAD4 and NOX, the presence of pathological H3cit neutrophils can be reduced, potentially providing a better understanding of the mechanisms underlying the mitigation of cerebral thrombosis [165] (Fig. 3).

Frederik et al. reported that neonatal neutrophils are incapable of forming NETs because of the presence of circulating NET inhibitory peptides (NIPs) [166, 167]. NIPs, which are cleaved pieces of alpha-1-antitrypsin, selectively inhibit NET formation, but other important neutrophil functions, such as phagocytosis or chemotaxis, are not affected [167, 168]. Administering neonatal NET inhibitory factor (nNIF) as a preventive measure in mice resulted in notable protection against the brain damage caused by ischaemic stroke. Mice were assessed through evaluations of neurological and infarct volume, motor function, long-term motor and neurological outcomes, and survival rate. The levels of both circulating and brain NETs were significantly decreased in animals treated with nNIF. Significantly, the decrease in NETs coincided with a notable decrease in cell death on the impacted side of the brain, indicating that NETs likely have dual effects on stroke by intensifying blood clotting in the brain and worsening damage to nerve cells [169].

The exacerbation of stroke and its impact on prognosis are attributed to brain inflammation caused by the recruitment of neutrophils [170]. To address immune dysregulation in the ischaemic brain, a sophisticated multifunctional delivery system has been developed. This system involves encapsulating PAD4 inhibitors within self-assembled liposomal nanocrystal carriers (C-Lipo/CA), which are further modified with polymers responsive to ROS and peptides that bind to fibrin. This modification enables targeted drug delivery to ischaemic lesions and facilitates controlled release. By regulating microglia, protecting the BBB, and promoting neuronal survival, C-Lipo/CA effectively mitigates the effects of ischaemia [59].

The efficacy of constraint-induced movement therapy (CIMT) in restoring motor function in ischaemic stroke patients has been substantiated by evidence-based medicine [171]. NETs primarily impair neurological function by disrupting the blood‒brain barrier and facilitating thrombosis [172]. Immunofluorescence and flow cytometry revealed the erosion of various areas, including the primary motor cortex (M1), striatum (Str), zona oblique vertical limb nucleus (VDB), zona oblique horizontal limb nucleus (HDB), and medial septal nucleus (MS), by NETs, which persisted within the brain parenchyma for a minimum duration of 14 days. However, CIMT reduced the levels of NETs and the chemokines CCL2 and CCL5 in M1. Interestingly, CIMT did not further reduce neurological deficits after inhibiting NET formation via pharmacological inhibition of PAD4 [173].

## Conclusions

After a stroke, neutrophils invade ischaemic brain tissue and can generate and release NETs through diverse mechanisms. NETs are strands of DNA found outside of cells that are made up of histones and granular proteins, which can trigger an inflammatory reaction, harm the endothelium of blood vessels in the brain, hinder the formation of new blood vessels after a stroke, and even mediate multiple cell death pathways. NETs can increase thrombosis through various mechanisms, exacerbate inflammation, and worsen stroke prognosis. Finally, NETs exacerbate postischemic brain injury and impede postinjury repair.

Inhibiting NETs mitigates the cerebral haemorrhage and BBB disruption caused by t-PA thrombolysis. This suppression possesses noteworthy potential for clinical advancement and serves as an objective for stroke treatment. Hence, the exploration of therapeutic strategies for stroke, such as the use of PAD4 inhibitors, DNase, and nNIF to target NETs, shows promise. Nevertheless, inhibiting NET formation may hinder the ability of neutrophils to eliminate pathogenic microorganisms and potentially have detrimental impacts on the immune system. Consequently, additional research is warranted to investigate methods for mitigating the adverse effects associated with NET-targeted therapy.

## Data Availability

No datasets were generated or analysed during the current study.

## Abbreviations

AIM2: Absent in melanoma 2
AIS: Acute ischaemic stroke
ANCA: Anti-neutrophil cytoplastic antibody
ATP: Adenosine triphosphate
BzATP: Benzoylbenzoic adenosine-triphosphate
cGAS: Cyclic GMP-AMP synthase
CLP: Caecum ligation and puncture
DCs: Dendritic cells
Dnase1: Deoxyribonuclease I
dsDNA: Double-stranded DNA
DVT: Deep vein thrombosis
EETs: Eosinophil extracellular traps
ERK: Extracellular regulated protein kinase
ET: Extracellular traps
FXII: Factor XII
GM-CSF: Granulocyte-macrophage colony stimulating factor
GSDMD: Gasdermin D
H3Cit: Histone 3 Citrullinated
HE: Haematoxylin-eosin
HMGB1: High mobility group box 1
HT: Haemorrhagic transformation
ICH: Intracerebral haemorrhage
IFN-β: Interferon-β
IL-1β: Interleukin-1β
IL-6: Interleukin-6
IL-17: Interleukin-17
iNOS: Inducible nitric oxide synthase
IRF3: Interferon regulatory factor 3
LETs: Lymphocyte extracellular traps
LPS: Lipopolysaccharide
LRP-1: Low-density lipoprotein receptor-related protein 1
Ly6G: Lymphocyte antigen 6G
MCETs: Mast cell extracellular traps
MEK: Mitogen-activated protein kinase
METs: Macrophage extracellular traps
MIP-1α: Macrophage inflammatory protein-1 alpha
MMP3: Metalloproteinase 3
MMP9: Metalloproteinase 9
MP: Microparticles
MPO: Myeloperoxidase
mPTP: Mitochondrial permeability transition pore
mtROS: Mitochondrial reactive oxygen species
NADPH: Nicotinamide adenine dinucleotide phosphate hydrogen
NE: Neutrophil elastase
NETs: Neutrophil extracellular traps
NF-κB: Nuclear factor kappa B
nNIF: Neonatal NET-inhibitory factor
NIPs: NETs-inhibitory peptides
PAD4: Peptidylarginine deaminase 4
PKC: Protein kinase C
PKM2: Pyruvate kinase muscle 2
PMA: Phorbol myristate acetate
PROG: Progesterone
PS: Phosphatidylserine
PTBK1: Phosphorylated TANK-binding kinase 1
P2X7R: P2X7 receptor
RA: Rheumatoid arthritis
RAGE: Glycation end products
PAR: Poly ADP-ribose
ROS: Reactive oxygen species
SLE: Systemic lupus erythematosus
STING: Stimulator of IFN genes
TBK1: TANK binding kinase 1
TF: Tissue factor
Th17: T helper 17
TLR2: Toll-like receptor 2
TLR4: Toll-like receptor 4
TLR8: Toll-like receptor 8
tMCAO: Transient middle cerebral artery occlusion
TNF-α: Tumour necrosis factor-α
tPA: Tissue plasminogen activator
vWf: Von Willebrand factor
